# Static Metabolic Bubbles as Precursors of Vascular Gas Emboli During Divers’ Decompression: A Hypothesis Explaining Bubbling Variability

**DOI:** 10.3389/fphys.2019.00807

**Published:** 2019-07-11

**Authors:** Jean-Pierre Imbert, Salih Murat Egi, Peter Germonpré, Costantino Balestra

**Affiliations:** ^1^Divetech, Biot, France; ^2^Department of Computer Engineering, Galatasaray University, Istanbul, Turkey; ^3^DAN Europe Research Division, Divers Alert Network (DAN), Roseto, Italy; ^4^Centre for Hyperbaric Oxygen Therapy, Military Hospital Brussels, Brussels, Belgium; ^5^Environmental, Occupational and Ageing Physiology Laboratory, Haute Ecole Bruxelles-Brabant (HE2B), Brussels, Belgium

**Keywords:** diving, decompression sickness, desaturation, oxygen window, pre-conditioning

## Abstract

**Introduction:**

The risk for decompression sickness (DCS) after hyperbaric exposures (such as SCUBA diving) has been linked to the presence and quantity of vascular gas emboli (VGE) after surfacing from the dive. These VGE can be semi-quantified by ultrasound Doppler and quantified via precordial echocardiography. However, for an identical dive, VGE monitoring of divers shows variations related to individual susceptibility, and, for a same diver, dive-to-dive variations which may be influenced by pre-dive pre-conditioning. These variations are not explained by currently used algorithms. In this paper, we present a new hypothesis: individual metabolic processes, through the oxygen window (OW) or Inherent Unsaturation of tissues, modulate the presence and volume of static metabolic bubbles (SMB) that in turn act as precursors of circulating VGE after a dive.

**Methods:**

We derive a coherent system of assumptions to describe static gas bubbles, located on the vessel endothelium at hydrophobic sites, that would be activated during decompression and become the source of VGE. We first refer to the OW and show that it creates a local tissue unsaturation that can generate and stabilize static gas phases in the diver at the surface. We then use Non-extensive thermodynamics to derive an equilibrium equation that avoids any geometrical description. The final equation links the SMB volume directly to the metabolism.

**Results and Discussion:**

Our model introduces a stable population of small gas pockets of an intermediate size between the nanobubbles nucleating on the active sites and the VGE detected in the venous blood. The resulting equation, when checked against our own previously published data and the relevant scientific literature, supports both individual variation and the induced differences observed in pre-conditioning experiments. It also explains the variability in VGE counts based on age, fitness, type and frequency of physical activities. Finally, it fits into the general scheme of the arterial bubble assumption for the description of the DCS risk.

**Conclusion:**

Metabolism characterization of the pre-dive SMB population opens new possibilities for decompression algorithms by considering the diver’s individual susceptibility and recent history (life style, exercise) to predict the level of VGE during and after decompression.

## Introduction

Hyperbaric exposures such as SCUBA diving are associated with a risk of decompression sickness (DCS). Traditionally, this risk has been attributed to the presence and quantity of decompression bubbles in the blood (vascular gas emboli or VGE) causing blood flow occlusion in various tissues. Recently, DCS has also been associated to the presence of blood microparticles and some symptoms linked to an inflammatory process (Thom et al., 2011; Arieli et al., 2015; Spisni et al., 2017).

In order to limit the risk of DCS, decompression procedures have been developed, as early as from 1908 (Haldane’s first experiments and publication of “dive decompression tables”). Although most diving operations are still conducted with empirical models, there has been a continuous research for editing safer decompression tables based on more realistic algorithms (Hugon, 2014). Various models have been developed and tested, and some have been implemented in diving decompression computers like the Bühlmann ZHL16 algorithm.

The objective of these models is to avoid or reduce bubble formation. However, there is evidence that bubbles are present in most, if not all decompressions, without necessarily representing a threat for the diver (Papadopoulou et al., 2013, 2015). VGE have been detected during decompression by *trans*-thoracic ultrasonic Doppler since 35 years. These bubbles have been estimated to be of 50 μm or larger size, in order to be detectable (Spencer, 1976). Graded levels of bubble detection have been used as an indication of decompression stress and, indirectly, risk of DCS (Nishi et al., 2003). A more sensitive method of monitoring is based on 2D echocardiography and uses actual bubble counts as an indication of the decompression stress (Germonpre et al., 2014). However, the resolution of the standard B-mode echography technique remains limited to roughly 35 μm (Papadopoulou et al., 2014, 2017), and it is reasonable to accept that in many divers, undetectable smaller precursor bubbles are present. The variability observed in levels of VGE measured in different divers, even when performing exactly the same dive profile, suggests that these precursors play an essential role in VGE production (Papadopoulou et al., 2018).

While current technology does not allow the direct detection of the precursors of these large vascular (venous) bubbles, their existence has been proven by indirect experiments in shrimps (Evans and Walder, 1969) and rats (Vann et al., 1980). These precursors were named “gas micronuclei” and estimated to be the size of 50–100 nm (Arieli et al., 2002). Linking these micronuclei to actual VGE detected in decompressing divers is still a matter of debate and requires several steps (Blatteau et al., 2006; Doolette, 2019).

The first step deals with cavitation in physical systems. Hills showed that cavitation occurs at the liquid/liquid interface after decompression when one of the liquids is hydrophobic (Hills, 1967). Recently, Arieli established that nanobubbles form on flat hydrophobic surface of silicon wafers from dissolved gas (Arieli and Marmur, 2011) and that these nanobubbles expand and detach to form free-floating bubbles after decompression (Arieli and Marmur, 2013a; Papadopoulou et al., 2015). This establishes a link between stationary nanobubbles on the blood vessel wall and blood-borne bubbles which prepares the scenario for decompression VGE.

The second step introduces cavitation in biological systems. Hills studied various endothelial surfaces from sheep and humans for their hydrophobicity, using a method based on the angle of contact, and found distinct hydrophobic areas (Hills, 1992). He concluded that the oligolamellar surfactant lining and lamellar bodies were potentially important factors in influencing bubble formation on vessel walls. Similarly, Arieli showed that the production of bubbles after decompression of ovine blood vessels is associated to active hydrophobic spots (AHS) that stain for lipids (Arieli and Marmur, 2013b, 2014) and confirmed that these AHS consist of deposit of hydrophobic lipids similar to or even originating from lung surfactant (Arieli, 2015).

Arieli was then able to visually observe the whole dynamics of bubble growth, detachment and the rate of bubble production. He derived a mathematical equation for conditional detachment which he based on buoyancy, and therefore, on reaching a critical bubble volume (Papadopoulou et al., 2015; Arieli and Marmur, 2017). He concluded that decompression bubbles in divers can develop only from pre-existing gas micronuclei (Arieli and Marmur, 2017), and that these could be nanobubbles appearing on active hydrophobic spots (AHS) as found on the endoluminal surface of blood vessels.

Studies on diver pre-conditioning have confirmed that stable stationary bubbles are most probably already present in the diver before the dive. This pre-existing bubble population can be affected by vibrations (Germonpre et al., 2009), exercise (Dujic et al., 2004; Castagna et al., 2011), sauna (Blatteau et al., 2008), and oxygen breathing (Castagna et al., 2009) before the dive. This population can also be modified by drugs that change endothelial function (Wisloff et al., 2004). All these pre-conditioning protocols reduce bubble formation and/or growth as demonstrated by reduced VGE levels measured after the dive. These experiments raise two questions:

• How could a small gas phase exist and remain stable during the ordinary life of a person before he becomes involved in a dive?
• How can factors such vibrations, exercise, heat, oxygen breathing, and endothelial-modulating drugs interfere with the bubbling process?

Recently, we showed (Balestra et al., 2016b; Germonpre and Balestra, 2017) that pre-dive vibrations better protect the diver than pre-dive oxygen breathing in terms of post-dive VGE count. We also observed that preconditioning with oxygen breathing before or during vibration is less efficient than vibration alone.

In this paper, we attempt to define a coherent system of assumptions that can:

• Describe a stable gas phase compatible with the properties of pre-existing bubbles involved in diver preconditioning experiments,
• Explain the differences observed between various pre-conditioning protocols.

For this purpose, we derive a simple mathematical model for the stability of such gas phase that provides an equilibrium equation based on local tissue metabolism. We then test the resulting equation against our previously published data (Balestra et al., 2016b) and the general literature to validate its predictions.

## Methods

Our theoretical approach is based on two identified dimensions of the problem, mechanical action related to vibrations, and metabolism, which we suspected was physically acting behind the scene through the “tissue inherent unsaturation” (Hills and LeMessurier, 1969; Hills, 1970).

To fill the gap between *ex-vivo* experiments and actual dives monitoring, we introduce metabolism as a parameter, characterizing any living organism. We postulate that metabolism can sustain a population of small gas pockets in the diver’s tissues before he starts his dive. We give those gas pockets the name of “Static Metabolic Bubbles” or SMB.

When considering these hypothetic SMB, the first problem is the stability of such gas phases. In a stable situation, because of surface tension, there must be a pressure difference between the sum of the gas partial pressures in the gas phase and the surrounding (ambient) pressure. The source for this pressure difference must be identified and quantified because it bears implications on the size of the gas phase and its possible evolution during decompression.

The second problem is the shape of this gas phase and the computation of its interfacial energy. Laplace’s law can only be used in configurations, where explicit curvatures can be defined, like for a sphere or a conical crevasse (Chappell and Payne, 2007), a characteristic that cannot be assumed for these biological gas pockets – of unknown shape. An alternative approach must be defined to account for surface tension.

### The Condition of Stability

We start with a stabilization condition for the initial metabolic bubbles. Any gas phase in a tissue of a diver at the surface should contain an inert gas (nitrogen), metabolic gases (oxygen, CO_2_) and water vapor. The stability of this gas phase first depends on the dynamics of gas exchanges through its surface. Because the diffusivity of metabolic gases is high, the gas exchanges are rapid (Van Liew, 1962) and we can reasonably assume that the gases in the bubble are in equilibrium with the adjacent medium. We define the adjacent medium as the venous side of the tissue as all the studies that could analyze gas concentrations in decompression bubbles have measured values close to the venous ones (Ishiyama, 1983; Foster et al., 1998).

The rationale for the stability equation is based on the gas solubility in liquids described by Henry’s law. For a dissolved gas, at equilibrium, at constant temperature:

(1) $${\text{P}}_{\text{i}}={\text{k}}_{\text{i}}^{\text{H}}{\text{c}}_{\text{i}}$$

where P_i_ is the gas pressure above the liquid, c_i_ the gas concentration in the liquid and ${\text{k}}_{\text{i}}^{\text{H}}$, Henry’s constant for the gas in the liquid at body temperature. By convention, the dissolved gas tension inside the liquid is defined as ${\text{k}}_{\text{i}}^{\text{H}}{\text{c}}_{\text{i}}$.

In the presence of a gas phase, Henry’s law is written for each gas dissolved in a diver’s tissue, considered to be in equilibrium with venous blood, that has diffused inside a local gas pocket. The equation below, derived by Van Liew et al. (1993), relates the gas phase pressures (Pb) to the tissue gas tensions:

(2) $$\begin{array}{lll} {\text{P}}_{\text{b}} & = & {\text{P}}_{\text{v},{\text{ O}}_{2}}+{\text{P}}_{\text{v},{\text{ CO}}_{2}}+{\text{P}}_{{\text{H}}_{2}\text{O}}+{\text{P}}_{\text{v},{\text{ N}}_{2}}\\ & = & {\text{k}}_{{\text{O}}_{2}}^{\text{H}}{\text{c}}_{{\text{O}}_{2}}+{\text{k}}_{{\text{CO}}_{2}}^{\text{H}}{\text{c}}_{{\text{CO}}_{2}}+{\text{P}}_{{\text{H}}_{2}\text{O}}+{\text{k}}_{{\text{N}}_{2}}^{\text{H}}{\text{c}}_{{\text{N}}_{2}} \end{array}$$

Note that because CO_2_ also combines into ${\text{HCO}}_{3}^{-}$ to form an acid-base buffer system that maintains the blood pH, the CO_2_ concentration in the above expression only refers to dissolved molecules.

### The Inherent Unsaturation of Tissues

The stability of a gas phase requires a pressure equilibrium. At this point, we hypothesize that metabolism, a purely physiological variable, could be linked to the physical world of bubbles by introducing the oxygen window (OW) as defined by Behnke (1967), also referred to as the “inherent unsaturation of tissues” by Hills and LeMessurier (1969).

The OW is a concept based on the “behavior” of metabolic gases. In a living tissue, the concentration of oxygen decreases and the dissolved CO_2_ concentration increases, due to metabolism. In terms of dissolved gas concentrations, the process is balanced, as there is nearly a 1 to 1 ratio between oxygen consumption and CO_2_ production (this is the Respiratory Quotient, varying from 1 to 0.8 at rest, depending on individual factors such as age, activity and nutrition). In terms of dissolved gas tensions, there is a significant difference as Henry’s constant for CO_2_ is 20 times higher than the one for oxygen. The decrease in oxygen tension is much more important than the increase in CO_2_ tension. As a result, the sum of the gas tensions on the venous side becomes lower than on the arterial side.

This difference between the sum of the venous tissue gas tensions and the ambient pressure is called the OW. It quantifies the capacity of the body to take in, transport and deliver oxygen to the tissue for a given activity. Its computation involves:

• Central factors such as alveolar ventilation, respiratory quotient, cardiac output, hemoglobin concentration, Hb-O_2_ affinity and perfusion rate.
• Peripheral factors such as tissue local blood flow, capillary density, oxygen extraction, oxygen diffusion and tissue metabolism.

The OW is theoretically defined by the activity of the mitochondria. Because pressures of metabolic gases are difficult to measure at this level, the OW is more conveniently defined across the arterial and venous sides of the tissue, for which concentrations of metabolic gases are well documented. In this work, we define the OW as the pressure difference between the ambient pressure and the tissue venous gas phase pressure as in the above-mentioned Van Liew publication:

(3) $$\text{OW}={\text{P}}_{\text{atm}}-{\text{P}}_{\text{b}}={\text{P}}_{\text{atm}}-\left({\text{P}}_{\text{v},{\text{ O}}_{2}}+{\text{P}}_{\text{v},{\text{ CO}}_{2}}+{\text{P}}_{{\text{H}}_{2}\text{O}}+{\text{P}}_{\text{v},{\text{ N}}_{2}}\right)$$

The gas phase described in Eq. 3 is not at pressure equilibrium and must evolve toward a new state according to the second principle of thermodynamics. The ambient pressure and partial pressures of constituting gases being fixed, the only solution for the system to cope with the OW is to create interfacial energy.

### The Classic Thermodynamic Approach to Bubble Stabilization

In Gibbs (free energy) thermodynamics, the modeling of gas supersaturated liquids requires computing the interfacial energy. Authors have proposed models, where bubbles are stabilized by Laplace’s law, a skin of surfactant at the liquid/gas interface (Yount, 1979b), a diffusion barrier (Yount, 1979a), tissue elasticity (Goldman, 2010), or combinations of the aforementioned.

Using Laplace’s law to express the interfacial energy between the liquid and the gas phase requires defining the curvature of the interface. For the simple case of a spherical bubble equilibrated with venous tensions it is classically expressed as:

(4) $${\text{P}}_{\text{atm}}-{\text{P}}_{\text{v}}=\frac{2\gamma }{\text{r}}$$

where γ is the surface tension, r the bubble radius and P_v_ the bubble internal pressure.

For a spherical bubble attached to a flat substrate, where θ is the contact angle and α the base surface radius, it becomes (Yount equation):

(5) $${\text{P}}_{\text{atm}}-{\text{P}}_{\text{v}}=\frac{2\gamma \cos(\theta )}{\alpha }$$

Explicit mathematical solutions have also been proposed for a gas phase located in a conical crevasse (Harvey et al., 1944; Tikuisis, 1986; Chappell and Payne, 2006).

In theory, for more complex configurations, splitting the interface into smaller elements of known curvatures should allow applying the Laplace’s law by finite element calculations. In practice, the difficulty is to define a geometry, especially for small bubbles where the interfacial energy becomes pre-dominant. Large discrepancies have been reported between the predicted internal pressure of a nanobubble and the actual measurement in physical systems (Ohgaki et al., 2010). The differences are expected to be even worse in biological systems because of the complexity and variability of living organisms. We therefore looked for an alternative formulation of the interfacial energy, avoiding any geometrical description.

### The Non-extensive Thermodynamics Approach to Bubble Stabilization

Non-extensive thermodynamics was developed to describe the behavior of solid and liquid condensates at the nanoscale, without having to refer to geometrical considerations. We chose the concept because it replaces curvature by volume and greatly simplifies our approach. It also applies without restrictions to nanometric as well as micrometric systems. The difference between the internal and external pressures of the above gas phase can be written as (Turmine et al., 2004):

(6) $${\text{P}}_{\text{atm}}-{\text{P}}_{\text{b}}=\text{m}\tau \alpha \frac{1}{{\text{V}}_{\text{b}}^{1-\text{m}}}$$

where V_b_ is the volume of gas and α a coefficient related to the system. The coefficient τ is an intensive variable that characterizes the interface similarly to the surface tension. It can be negative or positive which means that the system can be stabilized by either a negative or positive pressure difference, similarly to inward or outward curvature in Laplace’s law. The coefficient m represents the “thermodynamic dimension” of the system and is smaller than one. It somehow describes the shape of the system. For instance, setting τ = γ and *m* = 2/3 allows turning the above expression back to Laplace’s equation for a spherical bubble.

### The Static Metabolic Bubbles Volume Equation

We combine Eqs 3 and 6 to establish the condition of stability and obtain an original equation linking the OW to the stabilization energy of the SMB.

(7) $$\text{OW}=\text{m}\tau \alpha \frac{1}{{\text{V}}_{\text{b}}^{1-\text{m}}}$$

The above condition of stability demonstrates that:

• Metabolism can stabilize small gas pockets through the tissue inherent unsaturation;
• Metabolism controls this initial gas pockets volume.

The only requirement is a site with favorable thermodynamic conditions to generate a nanobubble that will evolve into a stable SMB. This pre-existing SMB population in living organisms becomes a direct consequence of metabolism:

• Metabolism provides the inherent tissue unsaturation required to stabilize the SMB.
• Metabolism is a continuous process that can sustain this SMB population over time.
• Metabolism can regenerate the SMB if the conditions are changed, provided the hydrophobic sites are persisting (Wienke and O’leary, 2018).

### Linking Metabolic Gas Pockets and Vascular Gas Emboli

Once the diver enters a decompression state, the dynamic of gas exchanges will feed the SMB by diffusion from the adjacent tissue (Van Liew et al., 1993). The SMB will grow until it reaches a critical volume for bubble detachment and generate blood-borne bubbles. The level of VGE detected in the divers will therefore depend on three parameters:

(1) The density of active sites.
(2) The initial SMB volume; this will define the time it takes for an SMB to grow to its critical volume of detachment, and thus determine the delay for the first bubbles to appear.
(3) The rate of ascent that will create the diffusion gradients and define the rate at which SMB grow and VGE are produced.

## Results

This paper consists in developing the consequences of a mathematical hypothesis and does not bring any original experimental results. It, however, refers to a long series of experiments we conducted on divers pre-conditioning (Germonpre et al., 2009; Balestra et al., 2016b; Germonpre and Balestra, 2017) for which explanations will be presented in the discussion.

### Estimation of the Oxygen Window

**FIGURE 1:**
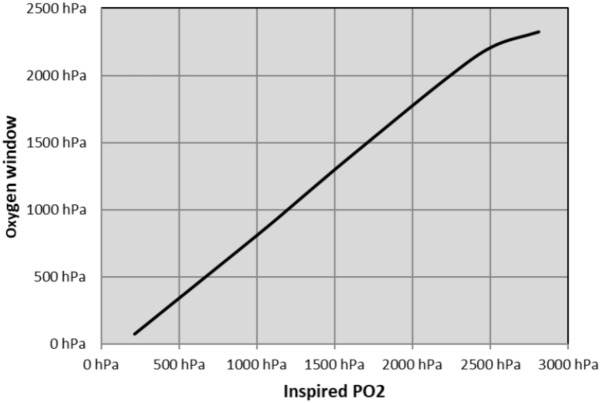
Oxygen window computed according to Egi’s model.

We calculated the theoretical OW based on Egi’s model (Egi, 1994). At surface, the inherent unsaturation is estimated at 70 hPa. At depth, tissue oxygen depends on the diver’s inhaled oxygen partial pressure. An increase of the pO_2_ in the breathing gas drastically influences the OW. This increase is linear until a point where the amount of dissolved oxygen is such that the tissue only consumes the dissolved oxygen and the hemoglobin remains maximally saturated. In this model, this point is achieved at around 2,200 hPa of pO_2,_ which corresponds to a diver breathing pure oxygen at a depth of 12 m. Beyond this point – which exceeds operational diving limits – the OW levels out at around 2,200–2,300 hPa (see Figure 1).

### Estimation of the Static Metabolic Bubbles Volume

Following the determination of the OW, Eq. 7 theoretically permits calculating the gas phase volume. However, the coefficients used in the formula cannot be defined numerically because of the lack of experimental data. To obtain an estimation, we adapted Eq. 7 to spherical bubbles by combining Eqs 4 and 6 to obtain:

(8) $$\text{OW}=\frac{2\text{γ}}{\text{r}}$$

This way, using a surface tension of 0.050 N.m^−1^ (Van Liew and Raychaudhuri, 1997) and two extreme values of the OW for diver breathing air at surface and a diver breathing pure oxygen at 12 m, we calculated a bubble radius ranging from 0.45 to 14 μm (Table 1).

**Table 1:** Estimated radius of spherical SMB for two diver situations.

	OW	Spherical SMB radius
Diver at surface breathing air	70 hPa	14 μm
Diver at 12 m breathing pure oxygen	2200 hPa	0.45 μm

The bubbles radii obtained are too large to be physiologically relevant. For a diver at surface, the bubble radius would correspond to 4 times the radius of a red blood cell and would not fit into a small blood capillary without seriously impairing the blood flow. This simply demonstrates that SMB are most probably not spherical and fully justifies our choice of Non-extensive thermodynamics based on volume rather than on radius.

## Discussion

### Limits to the Model: Sites for Static Metabolic Bubbles

Equation 7 is a static equation that does not carry any information on the initial growth of the SMB. It simply defines their stability once they have formed and reached a given size. It ignores the nucleation process and growth.

Equation 7 also does not bring any indication on the location nor the geometry of the sites (crevices, flat surfaces, and hydrophobic spots). It does not exclude any possibility either. It remains compatible with gas bubbles attached to hydrophobic surfaces on the vessel endothelium as described by Arieli.

With regard to the availability of these sites, we identify three parameters to characterize a given diver at surface, prior to a dive:

(1) The SMB population depends on the number of available sites for nucleation. For consistency with Arieli’s work, we considered hydrophobic sites located on the blood vessel endothelium. Therefore, for a given diver, we define a first parameter corresponding to the site density, that is the number of sites available per unit of tissue volume. This site density is a long-term characteristic that may evolve with age, as does metabolism (Cialoni et al., 2017).
(2) Because all the available sites may not be populated at all times, we further define the AHS, corresponding to sites actually occupied by a SMB. The effective AHS density depends on the recent history and condition of the diver, such as pre-conditioning interventions, or several dives with sufficient surface interval.
(3) The diver is finally characterized by the volume of his SMB population. According to Eq. 7, this volume depends on the tissue metabolism. It also depends on the inspired pO_2_ which much influences the magnitude of the OW.

For what concerns decompression, our understanding is that SMB could be present in any place that fulfils two conditions: a favorable physical site and enough tissue super saturation. Arieli observed in his first experiments that bubbles could generate from a silicon wafer, a purely physical support (Arieli and Marmur, 2011), but this required a high gas gradient created by decompression. In his following *ex-vivo* experiments, the tissues had no metabolism and the bubble formation remained purely physical even if they involved real tissues as a support. In divers, our mathematical derivation of the SMB stability considers the venous side of a tissue. However, it is admitted that SMB could form as well in the lymphatic vessels (Hugon et al., 2009; Balestra, 2014b) or the distal arterial tree (Arieli and Marmur, 2017).

### Limit to the Model: Size of the Static Metabolic Bubbles

The problem of identifying the gas micronuclei traditionally associated to decompression bubbles has recently been reviewed by Doolette (2019). He stressed the contradiction between the nanobubbles described in the physical chemistry literature and the physiological conditions of decompression, in particular, the extreme internal pressure calculated by Laplace’s law for a small spherical bubble. If the existence of SMB still remains hypothetical, their description avoids at least two pitfalls. First, they have no defined shape, just a volume, and therefore escape Laplace’s law (applicable only to a spherical bubble). Their interfacial energy can be much lower, as one could imagine when figuring for instance a tubular bubble. Secondly, their size is larger. These gas pockets are expected to have evolved to an intermediate volume set between the nanobubbles initially cavitating at the AHS and the critical volume bubbles that detach from these AHS to become VGE.

At surface, we could consider that various tissues in the diver’s body will have different metabolic rates. Therefore, prior to the dive, it would be reasonable to expect a distribution of initial SMB volumes rather than a single definite OW and volume of SMB. The formulation in Eq. 7 is therefore, a simplification.

During descent, the SMB volume will be reduced according to Boyle’s law. However, our assumptions do not allow us to decide whether there exists a crushing pressure for the collapse of the SMB as postulated by Yount (1979b).

### Limits to the Model: The OW Formulation

We have used Egi’s model of OW, where the role of CO_2_ is minimized. This is based on the fact that there is no “CO_2_ window” because CO_2_ dissolves into HCO_3_, a weak acid responsible for buffering the blood. The venous pCO_2_ is considered constant. On this basis, when the diver performs exercise, he increases his tissue metabolism and oxygen consumption, but the mathematical description shows that the OW remains almost unaffected because of the large availability of oxygen in the arterial side. With such a model, the influence of exercise on the tissue OW is small (a few hPa) at surface and is negligible in diving conditions up to 2000 hPa of inspired oxygen.

Other definitions of the OW exist depending on the assumptions considered. For instance, Kot et al. (2015) defined the OW using tissue metabolic gases values. This provided a higher estimate of the OW that they called the extended oxygen window.

Van Liew developed an improved OW model by taking into account CO_2_ variations with exercise (Van Liew et al., 1993) and the influence of CO_2_ on the oxygen dissociation curve. An increase of the P_v,CO2_ in Eq. 3 decreases the tissue OW. Consequently, according to governing Eq. 7, it results in a larger volume SMB. A high level of tissue CO_2_ could be the explanation to the findings of Wilbur et al. who detected, at surface, with a dual frequency ultrasound, a signal consistent with microbubbles after intense exercise on an ergonomic bicycle in a human subject’s leg (Wilbur et al., 2010) without any decompression.

Another improvement on the OW calculation was proposed by Walsh et al. who introduced the Michaelis Menten equation to describe the kinetic of oxygen uptake (Walsh et al., 2017). This relation allows computing a local non-linear oxygen consumption and accounts for the fact that different tissues may have different metabolism and therefore different OW’s. It could be the way to a more realistic computation of the OW based on a Gaussian distribution of tissue and individual metabolisms.

### Decompression: Relation Between Metabolic Gas Pocket and Vascular Gas Emboli Detection

We refer to the scenario proposed by Arieli and Marmur (2017), where the SMB are activated by decompression and grow until they release as a VGE in the blood stream, after the bubble has reached a critical volume of detachment. If buoyancy is the drive, it means that this critical volume of detachment is independent of the physical shape of the site and is a constant for every diver.

This scenario depends on the dynamic of gas exchanges because the surrounding tissue feeds the SMB, causing growth until it reaches its critical volume. After the first bubble has detached, the site will continue producing bubbles as long as gases will diffuse into it. This suggests that larger initial SMB detach more easily than smaller ones because they can reach this critical volume more rapidly (for the same local gas gradient). Therefore, the initial SMB volume is the first parameter controlling VGE once the diver is involved in decompression. This volume in turn depends on the tissue OW according to Eq. 7, which then refers to the diver’s metabolism. The interdependence between number of active spots and initial SMB volume could be the reason for the variability in VGE production observed between divers (Balestra, 2014a; Germonpre and Balestra, 2017; Papadopoulou et al., 2018).

### Variation of Metabolism: Influence of Age and Fitness on Vascular Gas Emboli Detection

Metabolism is known to decrease with age. According to Eq. 7, a decreased metabolism, associated to a reduced OW, should yield larger volume initial SMB. This could explain the higher levels of VGE detected in older divers (Carturan et al., 1999; Cialoni et al., 2017). Metabolism therefore introduces age as a first individual characteristic for a diver involved in a decompression.

Metabolism is known to be linearly related to cardiac frequency in rest (Kang et al., 2017). Also, the all-encompassing law of Kleiber has shown a linear relationship between the log of metabolism and the body mass (Kleiber, 1947). These factors suggest that fitness increases the OW (Painter, 2005). According to Eq. 7, an increased metabolism, associated to a higher OW, yield smaller volume SMB. A smaller gas phase takes a longer time to detach and produce fewer circulating bubbles during the decompression time. This would explain the lower levels of VGE observed in fit divers. Metabolism therefore introduces fitness as a second individual characteristic for a diver involved in a decompression.

The action of age and fitness are opposed and independent but can combine in each diver to produce variability. On one hand, an old and sedentary diver should have larger volume of initial SMB than a young and fit diver and produce more VGE. On the other hand, an old but fit diver could produce less VGE than a younger but sedentary diver.

In a recent large-scale database analysis, DAN Europe (Cialoni et al., 2017) concluded that only two factors, increased age and BMI, could be related to increased bubble formation. They also noted that neither height or weight separately had any relation to the bubbles. It is tempting to associate BMI to fitness for this divers’ populations because it would then provide the confirmation that because age and fitness act on the metabolism, they in turn acts on the OW and finally control the individual part in the bubbling process.

### Decreased Metabolism: Influence of Bedrest on Vascular Gas Emboli Detection

Confirmation of the above analysis is provided by a bedrest experiment recently published by Gennser et al. (2018). Authors used a 35 days’ bedrest conditioning to simulate microgravity and then ran several air dives to simulate extra vehicular activity. The dives were performed with divers still in bedrest conditions and were controlled by bubble Doppler monitoring. They concluded that 5 weeks of bedrest significantly increased bubble grades after decompression.

The analysis of this experiment using our model is coherent with the reported results:

• Bedrest conditions are associated to minimal activity and therefore to a minimal metabolism. The consequence is that the initial SMB volume in the divers prior to the dive was maximal according to Eq. 7.
• Then, the lack of exercise reduces vibrations and it is likely that most of the available AHS were populated by SMB.
• After a bedrest, the divers started the dive with a high density of SMB with a maximal volume that favored higher grades of detected VGE.

### Vibrations Preconditioning: Influence on Vascular Gas Emboli Detection

Preconditioning with vibrations produces a local energy release. The energy transferred will move the system to another equilibrium state. In this new state, changes in the internal pressure of the gas phase will modify its volume by either compression or temperature change and/or loss of gasses by diffusion. This will challenge its stability and the SMB may reach the critical volume and detach from its support. The main role of vibrations seems to reduce the number of AHS. The consequence of vibrations alone will be less VGE measured after decompression. However, vibrations do not affect the metabolism and therefore do not change the SMB volume according to Eq. 7.

**FIGURE 2:**
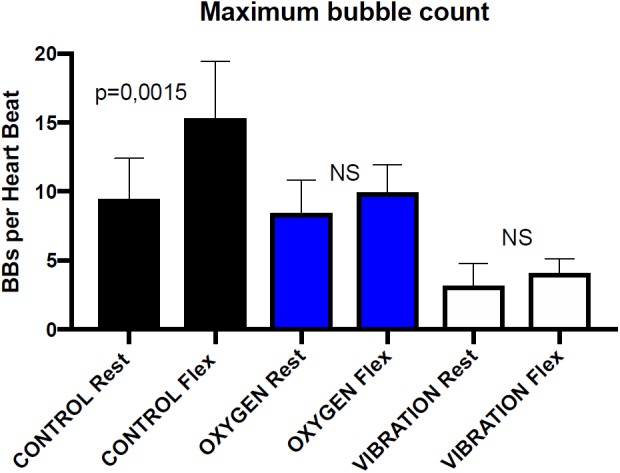
Maximal bubble counts before and after leg flexion using transthoracic echocardiography in 6 healthy divers known as consistent bubblers after a 33 m/20 min dive. The bubble maximal number is measured at 30 and 60 min post-dive. Both rest and post-flexion measurements are significantly lower with vibration pre-conditioning than with oxygen pre-breathing. Rest and post-flexion bubble counts are not significantly different in oxygen pre-breathing dives and in vibration pre-conditioning dives (Wicoxon matched-pairs signed rank test) redrawn from Balestra et al. (2016b).

Divers preconditioning experiments have shown that pre-dive vibrations reduce the number of VGE after decompression (Balestra et al., 2016b). Figure 2 shows VGE counts from this last paper, expressed in number of bubbles detected per heartbeat, at rest and after a leg flexion, for the control dives and the dives with pre-conditioning with vibrations and oxygen.

The protocol of VGE detections, with either Doppler or echocardiography, includes first a measurement at rest and then a measurement after a squat or leg flexions. The flexion generally triggers a rush of circulating bubble provoked by the muscular contractions. Referring to our model, it suggests that this flexion causes SMB to be dislodged from their sites before reaching the critical volume of detachment.

Data in Figure 2 shows that there is no significant difference between rest and flexion after vibration and oxygen preconditioning. For the vibration preconditioning, the explanation could be that SMB have already been dislodged before the dive, so the muscle contraction applies to a smaller number of SMB and produces less VGE (Germonpre and Balestra, 2017).

Data in Figure 2 may allow quantifying this protective effect in term of VGE count reduction, and may make it possible to define simple models and adjust coefficients by data fitting to predict the site density and initial SMB volume of a given diver or group of divers.

### Exercise Pre-conditioning: Influence on Vascular Gas Emboli Detection

Pre-conditioning with exercise is complex because exercise combines several effects. It increases metabolism and shear stress along the endothelium. It also produces a series of metabolic changes: vasodilatation, heat production, PCO_2_ increase and dehydration. Finally, it causes mechanical vibrations.

This principle of vibrations pre-conditioning from the above paragraph can be extended to exercise pre-conditioning if the metabolic response is ignored. The dose of vibrations obviously varies with the type of activity and this suggests that different exercises may have different efficiencies. For instance, running or mini-trampoline should be more efficient than swimming or cycling. Moreover, considering that the metabolism regenerates the SMB progressively, the frequency of the sport activity becomes important. In fact, the selection of the sport and the frequency of its practice define something we like to call an “healthy life style” (Thompson and Batterham, 2013), which has been suggested to be of importance to reduce diving DCS risk (Carturan et al., 1999, 2002).

### Oxygen Breathing Pre-conditioning: Influence on Vascular Gas Emboli Detection

Divers pre-conditioning experiments have shown that pre-dive oxygen breathing reduces the number of VGE after decompression.

Pre-dive oxygen breathing first involves de-nitrogenation and could protect the diver by reducing his tissue inert gas load before the dive. This protection is relative as it only concerns 790 hPa of surface PN_2_ compared to a bottom PN_2_ that could be five times larger.

According to our model, pre-dive oxygen breathing drastically increases the OW and reduces the SMB volume. Smaller volume SMB will take a longer time to grow and reach the critical volume for detachment and so will result in less VGE (Figure 2). This was confirmed by Van Liew who published that pre-dive oxygen breathing protects rats from decompression bubbles (Van Liew, 1998). However, pre-dive oxygen breathing is not expected to act on the density of SMB sites as vibrations would do (Blatteau et al., 2012).

### Combined Vibrations and Oxygen Breathing Pre-conditioning: Influence on Vascular Gas Emboli Detection

The results (Balestra et al., 2016b; Germonpre and Balestra, 2017) of the combination of vibrations and oxygen breathing in our pre-conditioning experiment showed that in term of post-dive VGE:

• Vibrations better protect the diver than pre-dive oxygen breathing.
• Combination of oxygen breathing before vibration is less efficient than vibrations alone.

Based on our model, these results can be explained by the following contribution of factors:

• On the one hand, vibrations reduce the initial population of SMB but do not change their size. Vibration is efficient in reducing the number of VGE detected after decompression.
• On the other hand, oxygen breathing increases the OW and according to Eq. 7, reduces the SMB volume. Smaller SMB will take a longer time to reach the critical detachment volume and will detach less easily. Pre-dive oxygen breathing is efficient in reducing the number of VGE detected after decompression.
• When combining the two above factors, if oxygen breathing comes first, the SMB will become smaller and more difficult to detach. The consequence will be that the same squeezing action (legs flexion) will detach only the biggest SMB after oxygen breathing than just alone keeping a higher number of VGE. The overall benefit will be less (Figure 2).

Our model suggests that oxygen breathing and vibrations can counteract their effects in this sequence. Alternatively, it proposes that the best pre-conditioning sequence would be to first expose the diver to vibrations to reduce the SMB density and then reduce the volume of the remaining SMB by oxygen breathing. However, this remains to be experimentally tested.

### Exercise or Stress During Decompression: Influence on Vascular Gas Emboli Detection

The situation is different for the previous discussion because change in the SMB population will occur during the dive. In addition, several factors will overlap: metabolism, cardiac function, heat/cold adaptation and exercise. The analysis therefore proceeds with simplification by separating the issues.

With regards to activity during or after the dive, it is understood that light exercise with little metabolic change but significant vibrations will release more circulating VGE according to our model predictions. However, two situations may occur.

In a first situation, if the ascent is badly controlled during decompression, or if the decompression table is inadequate, the excess of bubbles dumped into the venous bed might overload the lung and result in arterial bubbles and a higher risk of DCS (Madden and Laden, 2009).

On the other situation, provided the excess bubbles are filtered by the lung, exercise permits evacuating inert gas at a higher rate. Since there is more gas inside a bubble than dissolved in the same volume of blood, this results in a more efficient decompression. Dujic et al. reported that during SCUBA diving, light exercise at the 3 meters stop reduces post-dive VGE (Dujic et al., 2005) and that post-dive exercise induces a more rapid VGE decline (Dujic et al., 2006), indicating a faster decrease of tissue inert gas. Light exercise is therefore beneficial for a decompressing diver. For the same reason, commercial divers in saturation are encouraged to perform a daily session of light exercise during decompression.

With regards to stressful situations during the dive, such as cold or low visibility, they have shown to be statistically related to higher risk of DCS (Cialoni et al., 2017), even if depth and time of the dive were lesser. Stress by itself will trigger a series of physiological reactions such as cardiac frequency and ventilatory rate increase. It will also change the cellular oxygen consumption and metabolic rate as a response to stress and subsequent neuroendocrine reactions. The combined influence of these factors may be as yet not precisely calculable, but will (see Eq. 7) undoubtedly change the population of SMB, their localisation and behavior during decompression.

### Metabolism and Diet: Influence on Vascular Gas Emboli Detection

There is a renewed interest in nutrition in commercial diving to improve divers’ performances and eventually reduce their oxidative stress (Deb et al., 2016). It has been suggested that the diver’s diet has also a measurable effect on the level of VGE or on the vascular wall compliance which may also influence the SMB elimination (Theunissen et al., 2013, 2015; Valadao et al., 2014; Balestra et al., 2016a).

Metabolism is affected by nutrition. There is a possibility to link nutrition and metabolism through Eq. 7 because Egi’s model includes the respiratory quotient in the definition of the OW. This respiratory quotient could be used to link the diver’s diet directly to the VGE production.

### Implication of Static Metabolic Bubbles on Future Decompression Algorithms

The link between the presence of VGE and the risk of DCS is not direct. Even though Doppler monitoring has been used as the principal endpoint for the development and validation of most air tables (Spencer, 1976) and mixed gas tables (Nishi, 1991), large scale studies have demonstrated that:

• Vascular gas emboli may be present in any decompression without provoking any symptoms of DCS (so-called silent bubbles).
• There is a large variability among divers in term of VGE production (Papadopoulou et al., 2018).
• It remains that the lower the bubble count (or grade) the lesser the risk of DCS (Nashimoto and Gottoh, 1978; Gardette, 1979; Eftedal et al., 2007).

Linking VGE and the risk of DCS requires establishing a bubble scenario. In 1908, J. S. Haldane stated that “If small bubbles are carried through the lung capillaries and pass, for instance, to a slowly desaturating part of the spinal cord, they will there increase in size and may produce serious blockage of the circulation or direct mechanical damage” (Boycott et al., 1908). This scenario considers intravascular bubbles collected by the venous system and eliminated at the lung level in normal dive conditions. If a bubble passes into the arterial side, it may reach a tissue and causes local ischemia, mechanical damages and inflammation. The filtering capacity of the lung was first studied by Hills (Butler and Hills, 1979). Failure of the lung filter was proposed by James for the onset of CNS and spinal symptoms (James, 1982; James, 1991). Shunting of the lung filter explained the role of a patent foramen ovale (PFO) in the diver’s susceptibility to Type II DCS (Moon et al., 1989; Wilmshurst et al., 1989; Balestra and Germonpre, 2016; Lafere et al., 2017). Finally, Hennessy published in 1989 all the physical aspects of the arterial bubbles scenario in a paper that became the foundation of the arterial bubble assumption (Hennessy, 1989).

We have adopted this scenario to link VGE and the risk of DCS:

• The dose of incoming VGE and the lung filtration capacity determine the possibility of arterial bubble occurrence (Butler and Hills, 1979; Ljubkovic et al., 2012).
• The bubbles in the venous system trigger biological reactions with the vascular endothelium that create microparticles. These microparticles can pass in the arterial system and provoke a tissue inflammation similar to the one caused by bubbles (Thom et al., 2013, 2015; Yang et al., 2015).

We therefore consider that arterial bubbles and microparticles combine in the onset of DCS (Arieli and Marmur, 2017). The dose of incoming VGE produced by decompression remains the critical input to the lung filter.

The SMB assumption adds a step in the chain of events triggered by decompression. This intermediate step corresponds to a gas phase, located in the middle of scale, between nanobubbles nucleating at the AHS and micrometric or millimetric VGE dumped into the venous circulation.

Setting an acceptable level of decompression stress in a future algorithm will require defining four additional parameters. The first one will be estimate of the number and size of SMB, based on the diver’ individual characteristics. The second parameter will be the critical volume for bubble detachment that will control VGE production. The third will be the lung bubble filtration function that will determine the risk of passing arterial bubbles from the input dose of VGE (volume and number). The fourth one will be a risk function associating the number of arterial bubbles to the risk of DCS symptoms. Further improvements could consider the evolution of the number of sites and SMB volumes with repetitive diving and multi-days diving.

It makes it possible to supplement *M*-Values and gradient factors to decompression algorithms based on an initial SMB population adapted to the diver’s individual parameters.

The new algorithms will not necessarily produce much different decompression profiles. The current profiles were developed empirically but already provided an acceptable level of risk. Most instances of DCS, however, occur within the limits of these decompression profiles, for reasons as yet unaccounted for. This model could provide a better control of conservatism and offer the possibility to a given diver to select the level of decompression stress he is ready to accept for a given dive.

The pre-existing gas pocket population (SMB) therefore, becomes the diver’s main individual characteristic defining the VGE level measured during or after a decompression. It may also contribute to the other dimension of DCS, that is inflammation. Bubbles that detach from their support can strip apart the AHS as observed by Arieli (2017). The AHS may then reduce in size or even disappear after a series of bubbles detachments. Arieli used this possibility to explain divers’ acclimatization to intensive and repetitive decompressions. We consider that when VGEs are present, they could generate an inflammatory response because the endothelium would be physically altered by the “rubbing” effect of the VGEs. Causing some damage and retraction of endothelial cells, exposing the endothelial basement membrane to the blood and elicit interaction with inflammatory proteins (Blatteau et al., 2018).

## Conclusion

We postulated a pre-existing population of small static bubbles in divers, located preferentially on hydrophobic sites on the endothelial surface, populated and stabilized by tissue metabolism.

These gas pockets are expected to have an intermediate volume set between the nanobubbles initially cavitating at the AHS and the critical volume bubbles that detach from these AHS to become VGE.

We derived a stability equation linking the OW to the metabolic gas pocket volume without having to define any geometrical configuration.

We tested the assumption against our published experimental data and other relevant papers and found that pre-existing SMB:

• Are consistent with the observations on *ex-vivo* tissue bubble production by Arieli. This suggests that the level of circulating VGE depends on two parameters: the number of hydrophobic sites and the volume of the metabolic bubbles populating these sites.
• Can explain the results of diver pre-conditioning experiments using exercise, vibrations, oxygen breathing and combined vibration and oxygen breathing.
• Could explain why the individual variations of detected VGE in divers after decompression depend on the density of AHS and therefore seem to be linked to age and fitness.
• Suggest that physical activity type and frequency control this initial metabolic bubble population and therefore the level of VGE produced during a given decompression. In addition to specific individual factors, the diver’s “life style” could influence the observed variations in post-decompression VGE.

Our model links these small metabolic gas pockets to VGE measurements and the potential risk of DCS. This model offers two new possibilities for decompression algorithms (1) to adapt decompression to the individual factors of a given diver and (2) to select the level of acceptable decompression stress or DCS risk for a given dive.

## Author Contributions

J-PI and SME developed the hypothesis. PG reviewed the medical implications. CB developed the physiological part of the theory.

## Conflict of Interest Statement

J-PI was employed by company Divetech. The remaining authors declare that the research was conducted in the absence of any commercial or financial relationships that could be construed as a potential conflict of interest. The reviewer DC declared a shared affiliation, though no other collaboration, with one of the authors SME to the handling Editor.
